# Diabetes Self-Care in Older Adults With Type 1 Diabetes Mellitus: How Does Cognition Influence Self-Management

**DOI:** 10.3389/fcdhc.2021.727029

**Published:** 2021-09-13

**Authors:** Ana Lúcia Taboada Gjorup, Frank J. Snoek, Eelco van Duinkerken

**Affiliations:** ^1^ Post-Graduate Program in Neurology, Department of Neurology, University Hospital Gaffrée and Guinle, Federal University of the State of Rio de Janeiro, Rio de Janeiro, Brazil; ^2^ Department of Internal Medicine, University Hospital Gaffrée and Guinle, Federal University of the State of Rio de Janeiro, Rio de Janeiro, Brazil; ^3^ Department of Medical Psychology, Amsterdam University Medical Centers, Vrije Universiteit Amsterdam, Amsterdam, Netherlands; ^4^ Center for Epilepsy, Instituto Estadual do Cérebro Paulo Niemeyer, Rio de Janeiro, Brazil

**Keywords:** type 1 diabetes mellitus, cognition, self-management, dementia, diabetes care

## Abstract

With increasing knowledge and improvements in options for glycemic control, the life-expectancy of patients with type 1 diabetes mellitus (T1DM) has increased considerably over the past decades. Whereas this is undeniably positive for patients, aging is related to natural decline in cognitive functions. As patients with T1DM across the life-span are susceptible to cognitive deterioration, an interaction with aging may be expected and the risk of development of dementia might be increased. As achieving glycemic control depends on a set of diabetes self-management behaviors, it is imperative to understand how cognitive functions are involved in the upkeep of these behaviors and how cognitive impairment may affect them. In this narrative review, we set out to understand the relationship between cognition and T1DM self-care by first reviewing the glycemic targets in older adults, what treatment options are available, and what cognitive functions they draw upon. We will then review the cognitive literature in older adults that is available and then link both together. Lastly, we finish with clinical recommendations and suggestions for future research.

## Introduction

Over the past decades, treatment for type 1 diabetes mellitus (T1DM) has considerably improved, resulting in a delay in the occurrence and drop in prevalence and severity of microangiopathy, such as retinopathy (1), of cardiovascular disease (2), and an increase in life expectancy (3). Despite treatment advances, T1DM remains a behaviorally and cognitively demanding condition, highly dependent on the person’s motivation and self-management skills (4).

Although still lower than for the non-diabetes population (5), the life expectancy of T1DM patients has increased over the past decades. Ageing makes patients susceptible to cognitive impairment (6), which in turn may affect their diabetes self-care capabilities. Moreover, novel diabetes technologies, including continuous glucose monitoring and (hybrid) closed-loop systems generate complex glucose and insulin data, which patients need to evaluate and act on accordingly, increasing the information burden and cognitive demand of T1DM self-management (7).

There is increasing evidence that T1DM, across the life-span is related to cognitive impairment (8). In older adults, cognitive functions have been less frequently studied, but literature does suggest significant cognitive impairment (9), being related to cardiovascular disease, (severe) hypoglycemic events, and microangiopathy (9, 10). There is also a markedly elevated risk of vascular dementia in T1DM relative to controls, whereas the risk of Alzheimer’s disease is still under debate (11).

Given the increased risk of cognitive impairment in older adults with T1DM, one can question if and how these decrements impact on T1DM self-care capabilities and, consequently, on glycemic control. Unfortunately, literature on this topic is scarce. In this narrative review, we will explore the cognitive demands of the different treatments available for older adults with T1DM. We will then closely examine the profile of cognitive impairment in this group, and discuss studies that have related self-care to cognitive functioning. Lastly, clinical recommendations will be formulated with regard to diagnosis and management of cognitive impairment and T1DM treatment in this patient group.

## Treatment Options for T1DM and Their Cognitive Demand

Over the past decades, improvements in T1DM treatment, such as the development of long and rapid-acting insulin analogs and intensive insulin schemes, have allowed for improved glycemic control, while also controlling the risk of hypoglycemia (12). More recently, additional management strategies, including continuous glucose monitoring (GCM), closed loop and artificial pancreas systems, have been or are in development, which can further improve glycemic control. However, these technologies can be cognitively demanding in terms of data processing and decision-making.

### Current Practice and Its Cognitive Demands

Current best practice in T1DM treatment for older adults suggests intensive insulin therapy, with basal-bolus regimens, combining basal long-acting and prandial insulin or continuous subcutaneous infusions with insulin pumps. The aim is to achieve the best possible glycemic control, in terms of HbA1c and Time in Range (TIR), while preventing hypoglycemic events. However, successful treatment depends on the patient’s ability to adequately self-manage the diabetes, i.e. count carbohydrates, determine the amount of insulin necessary, act upon glucose levels outside of a determined range, and be aware of how much a unit of insulin decreases a person’s blood glucose level at any given time (13).

The Association of Diabetes Care and Education Specialists has proposed key competencies that are necessary for adequate diabetes self-care (14). They include healthy coping, healthy eating, being active, monitoring blood glucose levels, taking medication, problem solving, and reducing risks. Considering these 7 competencies, it is evident that T1DM treatment places a great demand on intellectual abilities and cognitive functions that may be compromised in older adults. When doubts arise about a person’s intellectual capabilities or cognitive functioning, an individualized assessment of cognitive competencies is useful to evaluate if a less complex regimen is called for. Less complex self-care strategies could include regimens with fixed pre-meal short-acting insulin doses or the use of premixed insulin (a combination of intermediate and rapid or short-acting insulins). The downside of these strategies is that the patient has to eat fixed amounts of carbohydrates, with little room for irregular eating or meal skipping.

In the case of moderate cognitive impairment or overt dementia, the responsibility of self-care shifts to the caregiver or medical professional, and may eventually have to be taken over completely from the patient. In these individuals, unpredictable meal intake can be an issue, thus, it is reasonable to maintain the basal-bolus insulin regimen, with a bolus dosing applied after the meal based on the amount of carbohydrate intake. With this approach it is possible to adapt the amount of insulin to the actual intake, preventing hypoglycemia in case the person eats less than expected (13). Another possibility is the use of a prandial bolus with an ultrafast-acting insulin analog, which can be administered up to 20 minutes after a meal, avoiding the postprandial glucose peak (12). Lastly, CGM devices with hyper- and hypoglycemia alarms can transmit data to caregivers, making it easier to monitor a patient’s glycemic control and recognize if they are at risk of dysglycemia (13).

### Other Treatment Options and Its Cognitive Demands

As mentioned, advancements in T1DM treatment options include the use of CGM, and hybrid closed loop systems, while most recently fully automated ‘artificial pancreases’ are entering the market but are not yet available in most countries. Some studies have found that the use of more advanced systems for self-care significantly improves HbA1c, where others did not (15). Time in Range (TIR), on the other hand, almost always seems to improve, thus leading to less episodes of hyper- or hypoglycemia (15). For older patients with T1DM, the International Consensus Report on TIR defined a target of 50% or more of the day spent in range between 3.9 – 10 mmol/l (70 – 180 mg/dl), compared to >70% in younger patients (16). The time below range [<3.9 mmol/l (<70 mg/dl)] was set to less than 1% of the day, whereas the time above range [>13.9 mmol/l (>250 mg/dl)] should be less than 10% of the day (16). This highlights the current guidelines of avoiding hypoglycemia. Improved TIR has been found in the DCCT trial to be directly associated with lower incidence of microangiopathy (17), and provides more clinical diagnostic information than just HbA1c for self-care regimen decision-making (18).

Although these technical advances provide great opportunities for older T1DM patients, they do require cognitive load, i.e. they draw on intact cognitive functioning, such as interpreting glucose information, integration of multiple self-care parameters, alongside the necessity to handle relatively complicated equipment. This may not be feasible for every older patient, especially when cognitive impairment is already present. If, however, with the right training and guidance by diabetes professionals, the patient is open to the use of such equipment, based on current literature, glycemic control may benefit greatly, lowering the risk of hypoglycemia. In the presence of already established cognitive impairment, CGM or other options may be of help if the caregiver is more involved, but these benefits should be weighed against simplifying the self-care regimen. Initially, the collected data should only be available to the patient, as it is their clinical data and theirs to share. With progression of cognitive impairment, however, caregivers or even health-care professionals could also have direct access.

## Cognitive Functioning in Older Adults With T1DM

In this review we will briefly focus on cognitive functioning in older adults with T1DM. For a detailed overview of cognitive functioning in younger patients please see a recent review (8). Studies including older patients with T1DM are scarce. A first study including 40 T1DM patients with a mean age of 61 years showed a mild decrement in processing speed of -0.34 standard deviation compared to controls (19). Generally, differences are considered clinically significant when they are -1.5 standard deviation or more below the mean of the reference group. After 4 years, 36 of the 40 patients were retested, without an indication of accelerated decline over time compared to the control group (10). However, it was demonstrated that patients with cardiovascular disease and those who have had severe hypoglycemic events during the follow-up period showed accelerated overall cognitive performance and processing speed decline (between -0.35 and -0.70 standard deviation) compared to their counterparts without any of these events (10). This study suggested that, although there does not seem to be a general decline in cognitive performance, specific groups of patients may be particularly vulnerable to cognitive decline.

The importance of hypoglycemia for cognitive decline is further demonstrated by the 32-year follow-up of the DCCT-EDIC cognitive data. They found that in the whole group decline between year 18 and 32 of follow-up was 5 times faster than between year 0 and 18 in psychomotor and mental efficiency, and memory, related to higher higher HbA1c, systolic blood pressure, and severe hypoglycemic events. Those with an updated HbA1c of ≥9.5%, a mean systolic blood pressure of ≥130, and >5 severe hypoglycemic events showed a decline of -2.82 standard deviation compared to baseline (20).

These data are in line with a study in a large group of 244 T1DM patients with a mean age of 55 from the Wisconsin Epidemiologic Study of Diabetic Retinopathy, demonstrating that recent severe hypoglycemia affected (approximately -0.35 standard deviation) processing speed and memory (21). In adults over 60 years of age with longstanding T1DM, hypoglycemia unawareness and recent severe hypoglycemic events were both independently related to higher levels of clinically significant cognitive impairment (9). In that study, clinically significant cognitive impairment, defined as 2 or more tests with a score of -1.5 standard deviation or lower compared to the reference group, was present in 48% of the 201 patients included (9). Interestingly, although cognitive decline over time seems limited, this is higher than the rate of 28% that has been found in middle-aged adults with childhood onset T1DM (22). Both studies also showed an influence of preexisting microvascular conditions and higher HbA1c on cognition, indicating it is not only hypoglycemia that drives cognitive impairment in older adults.

Although the number of studies on cognition in older adults with T1DM are limited, and study designs, patient populations, and neuropsychological tests used diverge between these studies, it has become clear that next to preexisting micro- and macroangiopathy and higher HbA1c, severe hypoglycemia can have a detrimental effect on cognition in older adults. This connects to the observations that severe hypoglycemia also negatively impacts cardiovascular health and that clinical care in older patients is aimed at preventing such events.

## Relationship Between T1DM and Dementia in Older Adults

The importance of the study by Chaytor et al. (9) assessing clinically relevant cognitive impairment, is that they used the same criteria as those for Mild Cognitive Impairment (MCI) (23). MCI, especially its amnestic form, is generally considered a precursor for dementia, in particular for Alzheimer’s disease. In determining the profile of clinically significant cognitive impairment Chaytor et al. showed that 12% of the group had a purely amnestic profile, compared to 44% showing a processing speed/executive functions profile, and another 44% having a combined profile (9). This may indicate that the risk of developing Alzheimer’s disease is relatively low. This hypothesis seems to be supported by a study based on national hospital registers combining a diagnosis of T1DM with a diagnosis of Alzheimer’s disease (11). The higher the age, the lower the risk of Alzheimer’s disease and in those 80 years and older, T1DM may even be protective (11). Whereas more studies specifically on T1DM are needed, it is reassuring that there is not a high risk of Alzheimer’s disease in patients with T1DM.

This seems to be different for vascular dementia, that is more related to vascular events and with slowing of mental speed. T1DM has been found to have a markedly elevated risk of vascular dementia, of 3.76 between the ages of 60 and 69, 2.09 between the ages of 70 and 79, and of 1.34 in those 80 and older (11). Similarly, the risk of any type of dementia is also markedly increased (24).

A recent study assessed the influence of the apolipoprotein E gene (ApoE) on cognition in middle-aged adults with T1DM. The ε4 allele of this gene is considered a genetic marker for the development of both Alzheimer’s disease and vascular dementia (25, 26). Results demonstrated that the presence of at least one ApoE ε4 allele negatively impacted speed-related cognitive functioning in patients, but not in controls (27). Diminishing speed of information processing is a hallmark of vascular cognitive impairment and vascular dementia (28). Memory or executive functions, domains often affected by Alzheimer’s disease, were not affected. This, although in younger patients, also supports the notion that older T1DM patients may be at a greater risk of vascular dementia, with the risk of Alzheimer’s disease being lower. Other risk factors of dementia in older adults with T1DM are relatively unknown. Contrary to type 2 diabetes, the presence of diabetic retinopathy was found not to be associated with dementia development over time (29). In another study, HbA1c over 8% has been related to a higher risk of developing dementia in T1DM (30). Interestingly, those with HbA1c levels between 6 to 6.9% and between 7 to 7.9% had a lower risk of dementia development.

More studies are needed in older adults to more precisely determine the time course of cognitive impairment and longitudinally establish the relationship between T1DM and dementia. Studies so far suggested that some groups of patients may be particularly susceptible to cognitive decline, although the contributors to a higher risk of dementia are largely unknown. From a cognitive perspective, it seems extra important to prevent severe hypoglycemic events in older adults with T1DM. This is in line with the current clinical literature which suggests that in older adults strict metabolic control does not have to be the objective. Rather, preventing (severe) hypoglycemia is considered more important. In the next sections, we will link cognitive performance to diabetes self-care in older patients.

## Evidence for an Interaction Between Cognition and T1DM Self-Care in Older Adults

There is a lack of studies assessing the relationship between cognitive functioning and T1DM self-care across all ages, and especially in older adults. Given that T1DM self-management depends on competencies, such as monitoring blood glucose levels and interpreting data to decide on necessary actions, taking medication, problem solving, and reducing risks, it is not difficult to understand that cognitive demands for self-care are high. In this context one can think of a number of cognitive functions, including arithmetic operations, executive functions (including e.g., planning), memory, and attention.

One study included 201 T1DM patients ≥ 60 years of age with a disease duration of 20 years or more (31). Diabetes numeracy was measured as an approximation of the quality of T1DM self-care, along with depression, instrumental activities of daily living, and neurocognitive functions. This study demonstrated that both memory and complex speeded attention were independently and positively related to diabetes numeracy, indicating that better functioning of these domains is related to better T1DM self-management. Moreover, the more pronounced the overall cognitive impairment, the lower were the T1DM self-care abilities (31). This is a clear indication based on observations in a large group of patients that cognitive functioning and deficits interact with self-care in older adults with T1DM. However, more research is warranted in order to better understand and map which cognitive functions are important for which domains of T1DM self-care.

## Suggestions for Clinical Practice and Future Research

### Clinical Practice

**Figure 1:**
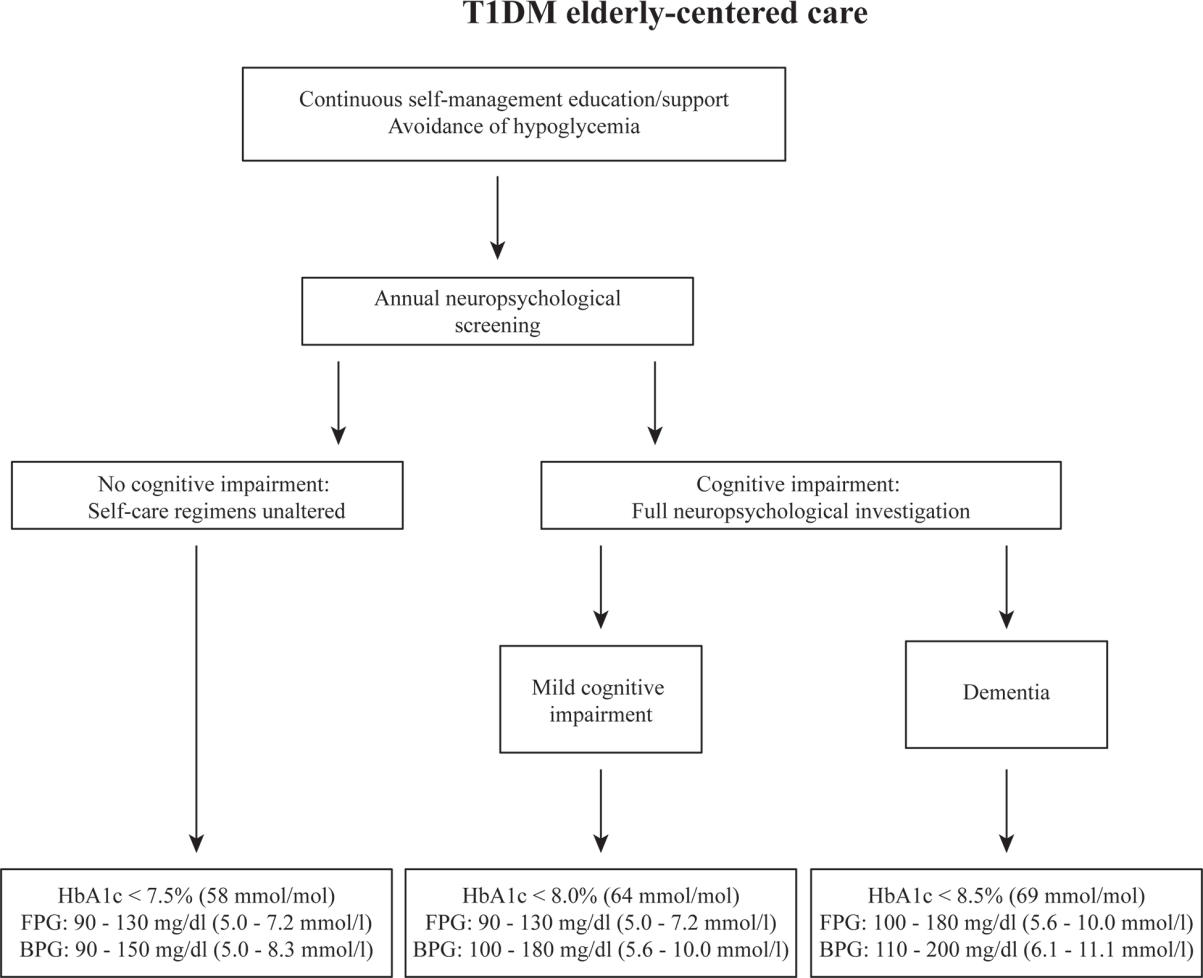
Flowchart of proposed steps to be taken in case older patients are suspected of cognitive impairment. The recommendations of clinical targets are based on the ADA’s clinical care standards. HbA1c, glycated hemoglobin; FPG, fasting blood glucose; BPG, bedtime blood glucose.

**Figure 1** shows a flowchart of how we envision diabetes (self-)care for older patients, including continuous diabetes education and neurocognitive testing as part of routine care. We also describe which glucose and glycemic control targets would correspond to what level of cognitive impairment. T1DM patients should receive self-management education and support as an integral part of patient-centered care (32). This should include a periodic assessment of educational needs and self-care skills, with a view on patients’ (changing) cognitive functions and competencies. Particularly in older adults, severe hypoglycemia can have deleterious effects in the short term, and also affect cognition in the long term and increase the risk of dementia. Prevention of hypoglycemia therefore is a priority, allowing for higher glycemic targets, if necessary, in this group of patients. The ADA standards for diabetes care in older adults suggest HbA1c levels below 7.5% in those with intact cognitive functioning, with fasting glucose between 5.0 – 7.2 mmol/l (90 to 130 mg/dl) and a bedtime glucose of 5.0 and 8.3 mmol/l (90 to 150 mg/dl). For patients with evident mild cognitive impairment, HbA1c should be below 8%, with similar fasting glucose levels, but slightly higher bedtime glucose levels (5.5 – 10.0 mmol/l [100 to 180 mg/dl]). In those with dementia, HbA1c targets are even higher, up to 8.5%, with a fasting glucose of 5.5 – 10.0 mmol/l (100 to 180 mg/dl), and a bedtime glucose of 6.1 – 11.1 mmol/l (110 to 200 mg/dl). We should of course recognize that beyond target HbA1c, minimizing glucose variability is of importance to avoid adverse effects on cognition and self-care, which may have implications for blood glucose measurement and medical therapy (33). The use of GCM in older T1DM patients can be a helpful tool to stay ‘safe’. It offers an Ambulatory Glucose Profile (AGP) report, showing TIR, and time above and below range over the past 7 days with a graphical, colored representation of the various patterns. Such reports could be simplified for older patients with cognitive difficulties and coupled with guidance from a health care provider and so-called if-then instructions in terms of timing and dosing of insulin and carbohydrate intake (16, 33). Highlighting the importance of cognitive status in older adults for glycemic targets it is evident that regular cognitive screening is imperative in this group. This could be a brief screening that provides information about the level of overall cognitive performance. It should be kept in mind, however, that most screening instruments, such as the Mini Mental State Examination or the Montreal Cognitive Assessment are designed to capture mild cognitive impairment and dementia of the amnestic/Alzheimer type and may not be adequate in detecting subtle cognitive impairment that can affect T1DM-related self-care behaviors. Therefore, a brief neuropsychological battery including tests for memory, working memory, complex attention and executive functions would be better suited. Granted this requires neuropsychological expertise and will take more time to execute, it will improve the validity of the screening and ability to draw conclusions about self-care abilities and the necessity to intervene. In older patients where there are serious concerns about cognitive abilities, either coming from the patient, their caregivers, or the diabetes professional, a full neuropsychological evaluation is required. This is also required when the brief assessment battery signals deficits in one or more tests. Based on the results of these assessments, the treatment regimen can be adjusted and self-care directed away from those behaviors that may be compromised. For example, when planning is affected, diabetes self-management could move to a more fixed insulin scheme requiring a regular eating behavior, removing the necessity for excessive planning. It is relatively common for diabetes clinics to not have neuropsychological expertise available. When part of a hospital, neuropsychological services are usually available, often as part of neurology, and a patient could be referred for diagnostic purposes. When not part of a larger hospital, patients could be referred to neuropsychological or memory clinics in the area. It is important, however, to have information exchange between diabetes health-care providers and a neuropsychologist for the best possible evaluation of cognitive functioning and cognitive load of specific daily self-care demands.

### Future Research

In terms of future research, there are 2 important lines of investigation warranting attention. The first line of research includes cognitive and dementia risk studies in the aging population of people with type 1 diabetes, linked to self-care behaviors. This will enhance our understanding of how cognitive impairment, and which specific domains of cognitive functioning over time impact on self-care behaviors and which behaviors are most affected. In addition to identifying which cognitive domains influence which self-care behaviors, it is also important to determine if and which ingrained (‘automated’) self-care behaviors used over the course of years are relatively protected against the effects of mild to moderate cognitive decline.

The second line of research builds upon these results and is focused on the development of effective programs and strategies that can assist to simplify T1DM self-care for patients, with the aim to preserve maximum patient autonomy. Such programs should aim at avoiding glucose extremes, but especially avoid (severe) hypoglycemia, as this has been directly linked to cognitive impairment in the older adult population. Of course, such programs can only succeed if patients are still able to learn new behaviors, which may be difficult in more advanced stages of cognitive decline. Research on these programs should include a taxonomy of ingrained, protected self-care practices, learning ability of the person living with T1DM and required level of support.

## Conclusions

In summary, type 1 diabetes self-care consists of a set of complex procedures that draws on intact cognitive functions, including complex attention, memory, and executive functions. In older adults, the glycemic targets are slightly more relaxed in order to avoid detrimental hypoglycemic events. However, in the case of cognitive decline over time, even highly learned self-management skills can be affected and deteriorated. Thus, it is imperative to evaluate the cognitive state of patients and execute a detailed neuropsychological assessment when there is suspicion of worsening glycemic control due to deteriorating cognitive functioning. Older adults with signs of cognitive decline should be offered a neuropsychological screening at least on an annual basis to objectively determine the level and speed of cognitive decline, already in the early stages. However, for screening to be most effective and sensitive, it is necessary to have a better understanding of which cognitive functions are involved in which self-management behaviors. Future research should focus on this link between self-care and cognition that informs the development of effective, patient-tailored programs that assist patients with self-care if needed. Taken together this ultimately needs to lead to preserved or improved glycemic control and quality of life, and, if necessary, to a smooth transition from independent to (partly) assisted self-care in those patients with cognitive impairment, while preserving maximum autonomy in the older adults.

## Author Contributions

All authors listed have made a substantial, direct, and intellectual contribution to the work, and approved it for publication.

## Conflict of Interest

The authors declare that the research was conducted in the absence of any commercial or financial relationships that could be construed as a potential conflict of interest.

## Publisher’s Note

All claims expressed in this article are solely those of the authors and do not necessarily represent those of their affiliated organizations, or those of the publisher, the editors and the reviewers. Any product that may be evaluated in this article, or claim that may be made by its manufacturer, is not guaranteed or endorsed by the publisher.
